# Pineal cyst apoplexy in a pregnant female: case report and review of literature

**DOI:** 10.1186/s12883-024-03922-7

**Published:** 2025-05-15

**Authors:** Mohammed A. Azab, Oday Atallah, Ahmed Hazim, Nour El-Gohary, Hamed Mostafa

**Affiliations:** 1 Department of Neurosurgery, Cairo University Hospital, Cairo, Egypt; 2 Department of Neurosurgery, Hannover Medical School, Hanover, Germany; 3 Department of Neurosurgery, Cairo University, Cairo, Egypt; 4 Department of Medicine, Cairo University, Cairo, Egypt; 5 Faculty of Medicine, Al Azhar University, Damietta, Egypt

**Keywords:** Pineal cyst, Apoplexy, Pregnancy, Headache

## Abstract

**Background:**

Pineal cyst is an uncommon condition in pregnancy. It is often encountered as an incidental finding. Most pineal cysts are benign and asymptomatic. Bleeding inside these cysts is rarely encountered in pregnancy.

**Clinical presentation:**

A 30-year-old female patient with no significant past medical history, presented to the emergency department at 36 weeks gestation with a transient episode of unilateral headache. Headache was associated with dizziness and left arm ascending numbness. She has a history of a known pineal cyst. The neurological examination was normal.

**Investigations:**

CT head was obtained, reviewed and compared to previous scans 7 years ago. It showed a hemorrhage inside the pineal cyst .

**Management:**

The patient was admitted to the neurosurgery department for conservative management and a few days later, symptoms gradually improved.

**Follow-up:**

Three weeks later, the patient reported spontaneous improvement of the presenting symptoms. The decision was to proceed with continued watchful follow-up and awaiting a caesarian delivery. The patient returned for a follow-up three months later without any symptoms. Following delivery, the patient remained asymptomatic.

**Conclusion:**

Pineal cyst apoplexy is a relatively rare condition and it usually affects young females, however, the exact relation to pregnancy and the effect of apoplexy on the course of pregnancy are not well defined.

## Introduction

Pineal cysts are common among females especially in the age of 20–30 [1]. Most of them are benign and their size ranges from 2 to 15 mm and they produce symptoms only when they reach a large size [2]. They rarely require treatment. The increased use of neuroimaging resulted in an actual increase in the prevalence of these cysts [3]. Pineal cysts are rare to occur during pregnancy. There is no documented relation between these cysts and certain pregnancy or endocrine problems, however, few studies reported that pineal cysts may affect the reproductive endocrinology [4, 5]. Robust animal and clinical research should be recruited to study the effect of pineal cysts on pregnancy, lactation and to study the effect of pregnancy on the behavior of these lesions.

We report an unusual case of pineal apoplexy during pregnancy presenting with migraine-like headache along with focal sensory problem. We also reviewed the literature to expose similar reported cases.

## Case Summary

A 30-year-old female patient with no significant past medical history, presented to the emergency department at 36 weeks gestation with an episode of transient throbbing headache.Headache was partially alleviated with over the counter analgesics. It was not associated with nausea or vomiting. Patient also complained of associated left arm ascending numbness. She does not have any history of neck pain, or blurring of vision.There is no history of past trauma, fever, chills or preeclampsia. She has a history of a known pineal cyst diagnosed 7 years ago. Since then, she was followed by other neurosurgeons without any complications or symptoms until her current pregnancy.

Patient was vitally stable, blood pressure was 120/70, and heart rate was 88. Neurological examination was normal. GCS was 15, motor power was intact in all limbs and reflexes were normal and symmetrical. Sensory examination of all the limbs was normal. Vision acuity was intact and no neck stiffness was observed on examination. Fundus examination did not reveal any abnormal findings. Cranial nerves examination was totally normal. The laboratory results for hormonal and coagulation profiles were within normal limits.

**Fig. 1:**
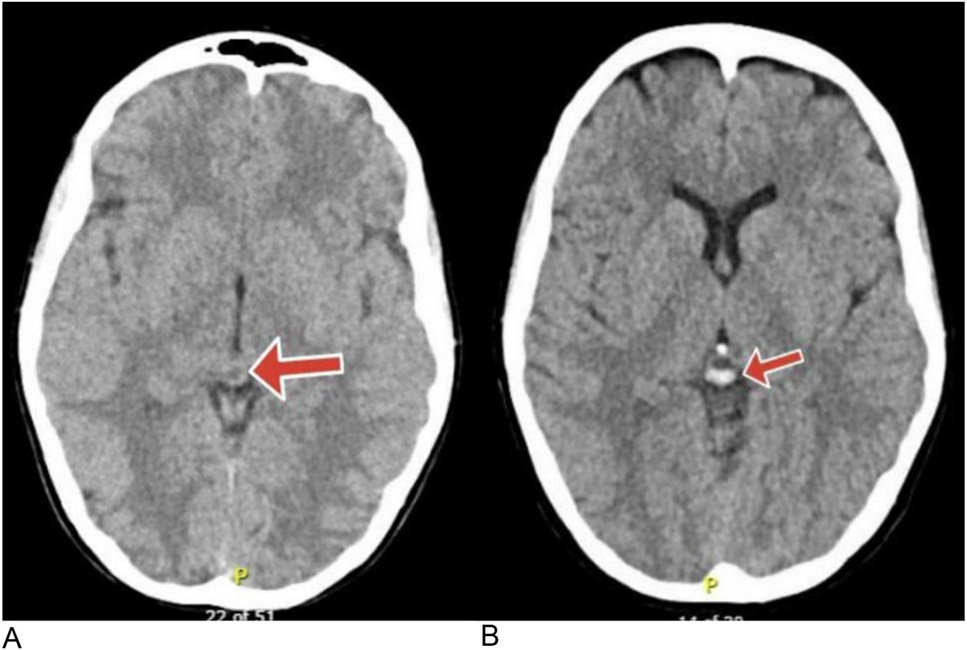
CT brain without contrast showing **(A)** Pineal cyst accidentally discovered seven years ago. **(B)** Hyperdense hemorrhagic blood products inside the pineal cyst

CT was obtained, reviewed and compared to previous scans 7 years ago. CT head showed a hemorrhage inside the pineal cyst without any evidence of hydrocephalus or brainstem compression. Figure 1(A,B). Susceptibility weighted images also revealed evidence of layered hemorrhagic components that appear as signal voids with a blooming effect in the pineal cyst and fluid-attenuated inversion recovery (FLAIR) MRI showed mixed intensity blood products inside the cyst. The patient was admitted at the neurosurgery department for observation and a few days later, symptoms gradually improved. Three weeks later, the patient reported spontaneous improvement of the presenting symptoms. The decision was to proceed with continued follow-up and awaiting a caesarian delivery. Three months later, the patient returned for follow-up without any symptoms. Following delivery, the patient remained asymptomatic and no further problems occurred over six months follow-up.

## Discussion

The pineal gland is an unpaired epithalamic neuroendocrine organ connected to the roof of the third ventricle [5]. The most common cell type is the pinealocyte which is a modified neuron with neuroendocrine properties that is responsible for secreting melatonin [5, 6]. Melatonin plays an important role in different physiological functions controlling sleep, wakefulness, and reproductive activity. Melatonin concentration in the ovarian follicular fluid is higher than in plasma with maximal concentrations reported in larger follicles [18]. Melatonin receptors were described in both granulosa and theca cells and activation of these receptors regulates progesterone production and female hormones receptor expression [19]. Additionally, oral administration of melatonin has been shown to improve fertilization rates in assisted reproductive techniques mostly due to melatonin’s antioxidant abilities [19]. Therefore, the role of the pineal gland in reproductive physiology is underscored.

The incidence of pineal cysts in young females may give a clue to a certain hormonal relation, however, the exact incidence of these lesions in pregnancy is unknown. The prevalence of these cysts ranges from 1.5 to 10.8% with a higher incidence in females confirmed by several retrospective studies [7, 12, 15]. The effect of pineal cysts on the production of melatonin and puberty is debatable. It has been reported that children with both precocious puberty and a pineal cyst have an unusually faster pubertal progress [5, 20]. In a study designed by Yuan et al. that included 4099 girls with central precocious puberty, the detection rate of pineal cysts was 6.4% which was significantly higher than that of other systemic diseases [24]. Several mechanisms have been postulated to explain a possible correlation between pineal cysts and reproductive problems. Pineal cysts may compress the hypothalamus affecting the release of gonadotropin releasing hormones [25]. The pineal cyst may alter the release of melatonin or may itself secrete gonadotropin like substances [25]. However, several studies disprove any relation of pineal cysts with abnormal puberty [21–23]. The association of pineal cysts with pregnancy is not well established due to the rarity of these lesions and the asymptomatic presentation. Also, pregnancy does not seem to affect the benign course of cysts accidentally discovered in the pineal gland .

Histologically, these cysts consist of an inner glial layer, a middle layer of pineal tissue, and an outer fibrous layer [2]. Pineal cysts are usually asymptomatic except when they acquire a large volume or in cases of apoplexy. They are usually stable and they rarely grow in size. Wisoff and Epstein described the clinical presentations of patients with symptomatic pineal cysts as either paroxysmal headache with gaze paresis; or chronic headache, papilledema, and hydrocephalus; or acute hydrocephalus resulting from pineal apoplexy [6]. Headache is a common symptom in pregnancy, the prevalence of secondary headache is about 14.3–52.6% among pregnant females [7].

Pineal cyst hemorrhage is an uncommon event and the incidence is unknown. Studies show that females are affected up to 3 times more than males, with peak occurrence in the third decade of life [2, 3]. We have described a case of a pregnant female who had frequent attacks of throbbing headaches and ascending upper limb sensory numbness. Posterior reversible leukoencephalopathy syndrome, reversible cerebral vasoconstriction syndrome, and preeclampsia are the main differential diagnoses in patients presenting with acute headache during pregnancy [7]. It is also essential to rule out cerebral venous thrombosis, idiopathic intracranial hypertension, subarachnoid hemorrhage, stroke, and pituitary apoplexy [8].

Unlike pituitary apoplexy, the factors that predispose to apoplexy in this location are not well defined, hormonal surges associated with pregnancy may be a contributing factor. Hypertension associated with pregnancy, the use of antiplatelet and anticoagulation increase the risk of apoplexy. A certain study suggested that a vascular malformation in the wall of the cyst may predispose to apoplexy [11]. Inherent fragility and poor structural integrity of the pineal vessels may also be a contributing factor. Histological examination of autopsy cases with pineal apoplexy revealed vessel hyalinization, and hemosiderin pigment deposition in the pineal layers [12]. Research to evaluate the risk factors for pineal cyst apoplexy should be advocated in animal and clinical models to promote our understanding of this clinical situation.

**Fig. 2:**
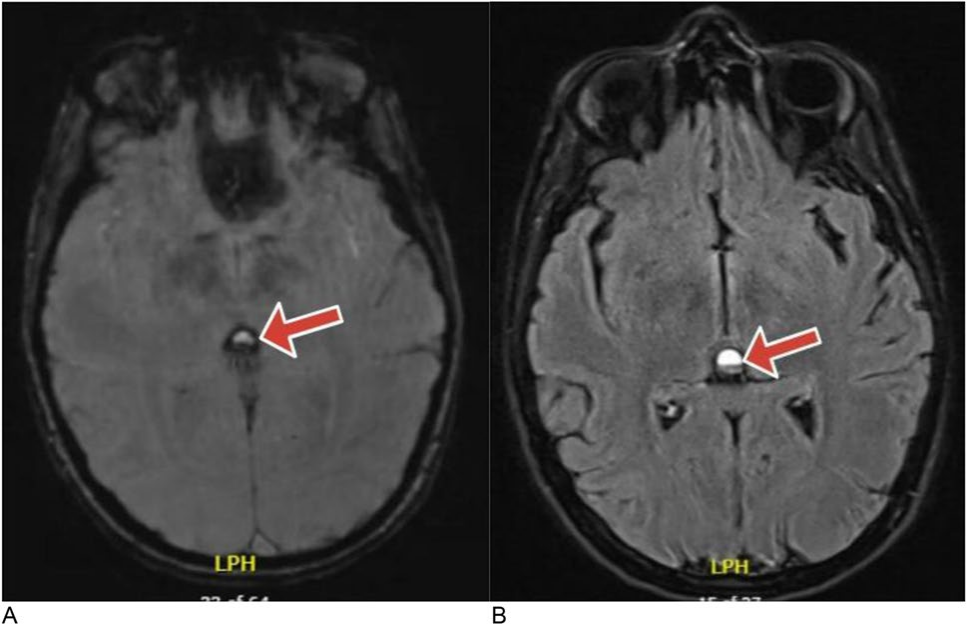
**(A)** Susceptibility weighted imaging (SWI) MRI without contrast showing an evidence of layered hemorrhagic components in the pineal cyst. **(B)** Fluid-attenuated inversion recovery (FLAIR) MRI showing mixed intensity blood products inside the pineal cyst

Based on the recurrent symptoms in this patient, it was necessary to perform imaging which showed apoplexy in pineal cyst. Apoplexy in this location may be completely asymptomatic or present suddenly with death [9]. The clinical presentations are variable and depend on the amount of bleeding that may vary from mild xanthochromia to frank subarachnoid or intraventricular hemorrhage [13]. The common presenting symptom is headache and it is characteristically, non-specific and may be associated with transient neurological deficits. Vision disturbances may be due to secondary hydrocephalus or direct compression of the midbrain [10]. Susceptibility weighted imaging (SWI) is sufficient to show the layering hemorrhage inside the pineal cyst Fig. 2. In certain cases, using CT scans may not reveal the apoplexy, therefore, performing MRI with increasing head tilt may be essential to show the blood fluid level especially in small hemorrhagic cysts [14]. In cases presenting with hydrocephalus, intracranial hypertension or brain stem compression, surgical intervention is essential. Aspiration or resection of the cyst are appropriate surgical options, however, it is challenging to determine the best treatment option. In asymptomatic patients without hydrocephalus, surgery could be deferred with proper follow-ups Table 1 [9, 10].

**Table 1:** Similar cases of pineal apoplexy during pregnancy

Authors	No of cases	Age	Gestational Weeks	Clinical presentations	MRI features	Management	Follow-up
**Cabral et al. **[17].	1	41	32 W	Acute right pulsatile hemicranial headache, vision disturbance	Internal blood–fluid level that was slightly hyperintense in T1-weighted and hypointense in T2-weighted/FLAIR MRI images, compatible with early subacute hemorrhage	Conservative management (blood pressure control)	Patient remained asymptomatic for one year.
**Our Case**	1	30	36 W	Acute unilateral headache, dizziness, ascending upper limb numbness	Fluid-attenuated inversion recovery (FLAIR) MRI showing mixed intensity blood products inside the pineal cyst. Figure 2A, B	Conservative management	Patient remained asymptomatic for six months of follow-up.

In our patient, given the absence of hydrocephalus, or brain stem compression and MR appearance of the cyst, we observed the patient regularly without surgical interventions. Spontaneous regression of the cyst was reported in certain studies [15, 16].

## Conclusion

Pineal cyst apoplexy is a relatively rare condition and it usually affects young adult women, however, the exact relation to pregnancy and the effect of apoplexy on the course of pregnancy is not well defined. In our patient, unilaterality of the headache, and the associated limb numbness are vague non-specific symptoms. There is no evidence about the role of surgery or conservative management in these cases.

## Data Availability

No datasets were generated or analysed during the current study.

## Abbreviations

CT: Computerized Tomography
MRI: Magnetic Resonance Imaging
